# *In silico* molecular docking and ADMET prediction of biogenic zinc oxide nanoparticles: characterization, and *in vitro* antimicrobial and photocatalytic activity

**DOI:** 10.1039/d4ra06890d

**Published:** 2024-11-12

**Authors:** Hajara Akhter, Susmita Sarker Ritu, Shahariar Siddique, Fariha Chowdhury, Rehnuma Tasmiyah Chowdhury, Samina Akhter, Mahmuda Hakim

**Affiliations:** a Biomedical and Toxicological Research Institute (BTRI), Bangladesh Council of Scientific and Industrial Research (BCSIR) Dhaka 1205 Bangladesh hajaragebcu@gmail.com hajara@bcsir.gov.bd; b Institute of Food Science and Technology (IFST), Bangladesh Council of Scientific and Industrial Research (BCSIR) Dhaka 1205 Bangladesh

## Abstract

Biogenic synthesis of metal oxide nanoparticles is a rapidly growing research area in the field of nanotechnology owing to their immense potential in multifaceted biomedical and environmental applications. In this study, zinc oxide (ZnO) nanoparticles (NPs) were biosynthesized from the *Citrullus lanatus* rind extract to elucidate their potential antimicrobial and dye degradation activity. The structural, morphological, and optical properties of the NPs were examined using various analytical techniques. UV-vis spectra showed a *λ*_max_ at 370 nm and the optical band gap was determined to be 3.2 eV for the ZnO nanocomposite. The FTIR spectrum denoted the functional groups responsible for the reduction of zinc acetate precursor to ZnO NPs. XRD demonstrated that the mean crystalline size of the nanocomposites was 20.36 nm while DLS, ζ-potential, FE-SEM, and EDX analysis of synthesized NPs confirmed their hydrodynamic size distribution, stability, morphological features, and elemental compositions, respectively. Biogenic ZnO NPs unveiled potent antimicrobial activity against *S. aureus*, *L. monocytogenes*, *E. coli*, *P. aeruginosa*, and *C. albicans*, showing 13 to 22 mm ZOI. This bactericidal activity of ZnO NPs was further elucidated using molecular docking analysis. The results showed a favorable lowest binding energy between ZnO NPs and microbial proteins (AusA for *S. aureus*, and CAT III for *E. coli*), which led to a possible mechanistic approach for ZnO NPs. Furthermore, the remarkable photocatalytic activity of ZnO NPs was revealed by the degradation of 99.02% of methylene blue (MB) dye within 120 min. Therefore, the above findings suggest that green synthesized ZnO NPs can be exploited as an eco-friendly alternative to synthetic substances and a unique promising candidate for therapeutic applications and environmental remediation.

## Abstract 2

A facile green route to synthesize zinc oxide nanoparticles using watermelon rind extract with antimicrobial and photocatalytic potential.

## Graphical abstract

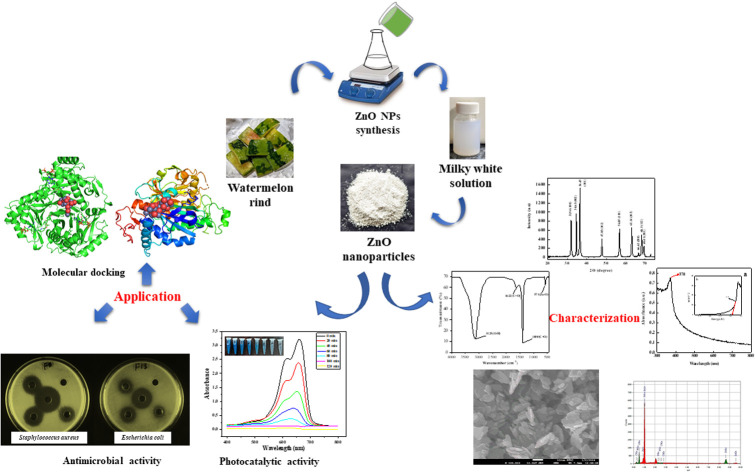

## 1 Introduction

The emergence of antibiotic resistance is a global public health challenge in which antibiotics are no longer responding to antimicrobial medicines. The abandoned use of antibiotics is the predominant cause of antimicrobial resistance (AMR) and has been estimated to have caused 1.27 million global deaths in 2019 which is projected to upsurge to 10 million by 2050 if no action is taken.^1,2^ The World Health Organization (WHO) has reported a caution that the world is “running out of antibiotics,” escalating the quest for alternative therapeutic approaches.^3,4^ In this context, nanoparticles (NPs) with antimicrobial potential offer promising avenues for facing critical periods of microbial infection. The therapeutic efficacy of nanoparticles is largely derived from their nanoscale size and highest surface-to-volume ratio, which enables multivalent interactions with target pathogens.^5,6^ Besides their use in therapeutics, these distinctive physicochemical properties have broadened their applications in a multitude of state-of-the-art fields, including electronics, agriculture, chemical catalysis, *etc.*^7^

Among various NPs, zinc oxide (ZnO) nanoparticles embrace ample aptitude due to their noteworthy physicochemical properties like tiny size, increased surface area, low melting temperature, and structural stability, along with their non-toxic and biocompatible nature.^8,9^ Additionally, owing to its distinct properties such as strong UV absorption capacities, wide band gap, excellent redox properties, and higher excitation binding energy, ZnO is recognized as an exceptionally potent photocatalyst, particularly in applications related to the photodegradation of organic dyes in wastewater treatment.^10^ The FDA characterized ZnO as a safe food additive (GRAS) with minimal toxicity.^11^ Hence, ZnO NPs employ extensive biomedical applications, including antioxidant, antimicrobial, antidiabetic, anticancer, drug delivery, *etc.*^12^

The ZnO NPs are synthesized using several chemical and physical methods, including electrochemical processes, hydrothermal methods, laser ablation, lithography, microwave-based synthesis, and thermal decomposition.^13–15^ However, these conventional approaches have many drawbacks, including strict pressure or temperature requirements, laborious procedures, expensive, time-consuming, and in particular perilous or hazardous chemicals that pose risks to the environment and human health.^16,17^ Hence, it is crucial to adopt alternative methods that are reliable, eco-friendly, energy-efficient, cost-effective, safe for human therapeutic use, and sustainable. The green method of NPs synthesis has gotten a lot of interest in the past decades and is considered an alternative approach involving the use of microorganisms, plants or plant parts, and biodegradable waste.^7^ Until now, different types of biological agents have been demonstrated to be employed in the efficient and successful synthesis of ZnO NPs having antimicrobial, anticancer, antidiabetic, antioxidant, larvicidal, wound healing, and photocatalytic properties.^16,18^ However, a comprehensive literature review reveals that very few studies have examined the potential of bio-based wastes, which are discarded in garbage, to be converted to value-added products, including metal oxide NPs.^19^ To date, different kitchen wastes such as orange peel^20^ banana/plantain peel,^21^ and pomegranate peel^22^ extracts were employed for the synthesis of ZnO NPs. Hence, the potential of other bio-based waste like *Citrullus lanatus* (watermelon) rind extract for the fabrication of zinc oxide nanoparticles (ZPCL) needs to be fully explored.

Watermelon is an herbaceous creeping or climbing plant of the Cucurbitaceae family. It is one of the most cultivated fruits in tropical and subtropical countries.^23^ The fruit is characterized as the largest, heaviest, and juicy fruit and composed of the fleshy red pulp (edible part), seed (thoroughly dispersed in flesh), and rind (waste part of the fruit which contributes approximately 30–40% of the total weight).^24^ The fleshy pulp and seed have nutritional value but watermelon rind (WR) is typically discarded as kitchen waste, causing biomass loss and environmental pollution.^25^ Numerous studies demonstrated that WR is rich in citrulline, fatty acids, minerals, dietary fibers, phenolic compounds, alkaloids, sterols, cucurbitacin, and triterpenes, *etc.*, has potent antioxidant properties.^26–28^ WR also contains cellulose, hemicelluloses, pectin, lignin, carotenoids, and phytates.^27,29^ Nevertheless, Bichi *et al.*, reported WR's aqueous extract demonstrates thirty-one (31) bioactive constituents including hexadecanoic acid, 2-hydroxy-1-(hydroxymethyl) ethyl ester, eicosane, 1-hexadecanesulfonic acid, 3,5-dichloro-2,6-dimethyl-4-pyridyl ester, 9,12-octadecadienoic acid(Z, Z)-2,3-dihydroxypropyl ester, Estra-1,3,5(10)-trien-17β-ol, *etc.* identified by GC-MS and FTIR analysis.^30^ It has been shown that these bioactive molecules and metabolites possess several functional groups like hydroxyl, carbonyl, and amine which are indispensable for reducing as well as capping/stabilizing the biosynthesized NPs.^29,31^ Recently very few studies have investigated the potential of ZnO NPs photocatalytic activity by degrading organic compounds (*i.e.*, metronidazole and methylene blue) synthesized from watermelon peel extract.^32,33^ However, to the best of the authors' knowledge, this is the first study to investigate the antimicrobial properties of ZnO NPs synthesized from watermelon (*C. lanatus*) rind that was further explained using molecular docking and ADMET prediction.

## 2 Materials and methods

### 2.1 Materials

Watermelon rind (WR) wastes were obtained from Jatrabari Kacha Bazar, Dhaka, Bangladesh, and were further systematically identified by a taxonomist of the National Herbarium of Bangladesh (DACB 99120). Zinc acetate dihydrate [Zn(CH_3_COO)_2_·2H_2_O], sodium hydroxide (NaOH), methylene blue (C_16_H_18_C_l_N_3_S) and ethanol (CH_3_CH_2_OH) were purchased from Sigma Aldrich, Merk, Germany. All the chemicals were of analytical grade.

### 2.2 Methods

#### 2.2.1 Preparation of the extract

Collected WR was cleansed properly with running tap water which was subsequently washed with deionized (DI) water to eliminate any contaminants. WR was cut into small pieces (5 mm × 10 mm) and placed into a conventional blender for crushing. A total of 100 g of the crushed WR paste was heated with 400 mL of DI water followed by the extraction under reflux conditions at 80 °C for 3 h. A light-green-colored WR extract was obtained after the filtration of the mixture through Whatman filter paper no. 1 and chilled in a refrigerator at 4 °C.

#### 2.2.2 Biosynthesis of ZPCL

**Fig. 1:**
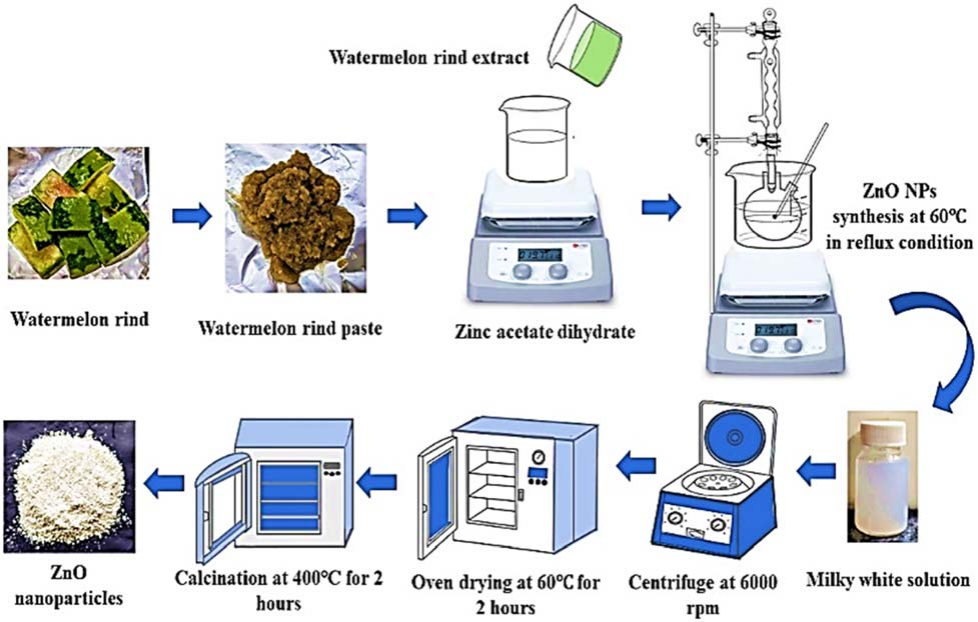
Schematic model of green synthesis of ZPCL using *C. lanatus* rind extract.

ZPCL were biosynthesized from *C. lanatus* rind extract following earlier reported literature with slight modifications.^34^ Briefly, about 0.1 M zinc acetate dihydrate was dissolved in 50 mL of DI water and the resultant mixture was swirled vigorously in a magnetic stirrer at room temperature until a clear solution was obtained. After that, 5 mL of aqueous WR extract was added to the zinc acetate solution and maintained at a pH value of 12 by slowly adding 1 g of NaOH. The mixture was heated at 60 °C for 4 h at reflux condition with continuous stirring. After the reaction reached its completion, the resultant milky white solution was allowed to precipitate and cooled to room temperature which was then centrifuged at 6000 rpm for 30 min. The supernatant was decanted, and the remaining precipitate after decanting was washed several times using DI water to remove any trace of impurities before oven-drying at 60 °C for 2 h. Following synthesis, the sample was calcined in a Muffle furnace for 2 h at 400 °C. Using a pestle and mortar the light white colored material found after calcination was ground into fine powder and stored in an airtight container for further physical characterizations and biological applications. The stages involved in biofabfrication of ZPCL from *C. lanatus* rind aqueous extract are presented in Fig. 1. The yield of ZPCL was estimated using the following formula:

1 Yield (%) = (Weight of ZPCL/Weight of ZnC_4_H_6_O_4_) × 100

#### 2.2.3 Characterization of synthesized ZPCL

The size, shape, crystallographic structure, functional groups, stability, and elemental analysis of the biosynthesized ZPCL were all determined using a variety of analytical techniques. These consist of UV-vis spectroscopy, fourier transform infrared spectroscopy (FTIR), energy dispersive X-ray spectroscopy (EDX), field emission scanning electron microscopy (FE-SEM), dynamic light scattering (DLS), zeta potential (ζ), and X-ray diffraction (XRD). UV-vis spectra were monitored in the wavelength range of 200–800 nm and the band gap energy (*E*_g_) of ZPCL was determined using Tauc plot. The crystalline structure and size of biosynthesized ZPCL were measured by an XRD with Cu Ka radiation (Rigaku Smart Lab, Japan).^35^ FTIR (PerkinElmer, USA) analysis was performed to detect the functional groups and phytochemicals helping in the reduction and stabilization of the NPs as well as the Zn–O bonds. The ζ potential of synthesized ZPCL was determined using nanoparticle analyzer (nano Partica SZ-100-S2, HORIBA scientific Ltd, Japan) to investigate the stability of nanoparticles dispersed in aqueous media. Particle size distribution have also been examined by DLS based nanoparticle analyzer (nano Partica SZ-100-S2, HORIBA scientific Ltd, Japan). The surface morphology and physical dimensions of biofabricated ZPCL were examined using an FE-SEM (JSM-7610F, Japan). ImageJ software was used to calculate particle size distribution from FESEM images. EDX was conducted to identify the elemental compositions of the biosynthesized nanoparticles.

#### 2.2.4 Antimicrobial assay

*In vitro* antimicrobial effects of biologically synthesized ZPCL were evaluated against various pathogenic bacteria and fungi using the agar well diffusion assay.^36^ The pure cultures used in the test were *Staphylococcus aureus* (ATCC 6538), *Listeria monocytogenes* (ATCC 13932), *Escherichia coli* (ATCC 25922), *Pseudomonas aeruginosa* (ATCC 9027), *Candida albicans* (ATCC 10342). The microorganisms were subcultured in a nutrient broth medium at 37 °C for 24 h. Approximately 5 × 10^5^ CFU (0.5 McFarland standards solutions) of each microbial strain was equally spread on Muller–Hinton agar (MHA) plates to induce sporulation. Then, 6 mm diameter wells were punched on the agar plates using a sterile cork borer, and different concentrations of the ZPCL solution (50, 100, and 200 μg mL^−1^) were placed on the well from the 1 mg per mL stock solution in 50% DMSO. Then the MHA plates were kept in an incubator at 37 °C for 24 h. Positive control was chloramphenicol (100 μg mL^−1^) and carbendazim (100 μg mL^−1^), and negative control was 50% DMSO.

#### 2.2.5 Molecular docking

To explore molecular insights and validate the observed antibacterial findings, the study evaluated the activities of ZnO NPs using molecular docking simulations. The present work retrieved 3D crystal structure of aureusimine biosynthetic cluster reductase domain (PDB: 4F6C, for *S. aureus* strain Mu50), chloramphenicol acetyltransferase type III (PDB: 6X7Q, for *E. coli*) as microbial enzyme from RCSB protein data bank (PDB). The ZnO hexagonal crystal structure was obtained from the materials project website whereas the 3D structure of chloramphenicol (CHL) was taken from PubChem as standard. All crystal structures of the proteins were prepared by removing hetero atoms, water molecules, and co-crystallized ligands using Discovery Studio Visualizer. Then the A chain was selected for analysis and subjected to energy minimization utilizing Swiss-PDB Viewer (4.1.0). Polar hydrogen atoms, Gasteiger partial charges, and Kolman charges were then added to the target molecules and ligands using MGL Tools 1.5.6.^37^ Docking was performed using PyRx (0.8) software considering the target protein as a macromolecule and ZnO NPs or CHL as a ligand by maximizing grid box size.^38^ The binding pattern that represented the highest negative energy was considered the best-docked model that was further used to visualize binding sites and calculate non-bonding interactions using the BIOVIA Discovery Studio v2021 software.^39^

#### 2.2.6 ADMET prediction

The AdmetSAR and PRO TOX-II (https://tox-new.charite.de/protox_II/) online server was used for screening *in silico* ADMET (absorption, distribution, metabolism, excretion, and toxicity) properties of ZnO NPs as drug candidate while CHL was used as standard drug.^17,40^

#### 2.2.7 Photocatalytic activity

To evaluate the photocatalytic potential of the NPs, biofabricated ZPCL was used as a photocatalyst and methylene blue (MB) as the model dye. In this experiment, 20 ppm of MB dye solution was prepared in 50 mL of DI water.^18^ An approximate 5 mL of MB solution was separated in a test tube for its UV absorbance recording. Then, 25 mg of ZPCL catalyst was mixed with the remaining dye solution. After vigorous shaking, the solution was subjected to incubation in a dark room for 20 min to attain adsorption–desorption equilibrium. Then, the mixture was exposed to sunlight, and the change in MB dye concentration was assessed at 20 minutes intervals. After this, the solution was centrifuged for 15 min at 9500 rpm to separate the photocatalyst from the dye solution. The decomposition of the dye was analyzed by a UV-visible spectrophotometer. The % dye degradation was estimated using the following formula

2 Dye removal efficiency (%) = [(*C*_0_ − *C*_*t*_)/*C*_0_] × 100

where *C*_0_ and *C*_*t*_ are the initial and the final concentrations at different time intervals of MB dye, respectively.

## 3 Results and discussion

### 3.1 Biosynthesis of ZPCL

**Fig. 2:**
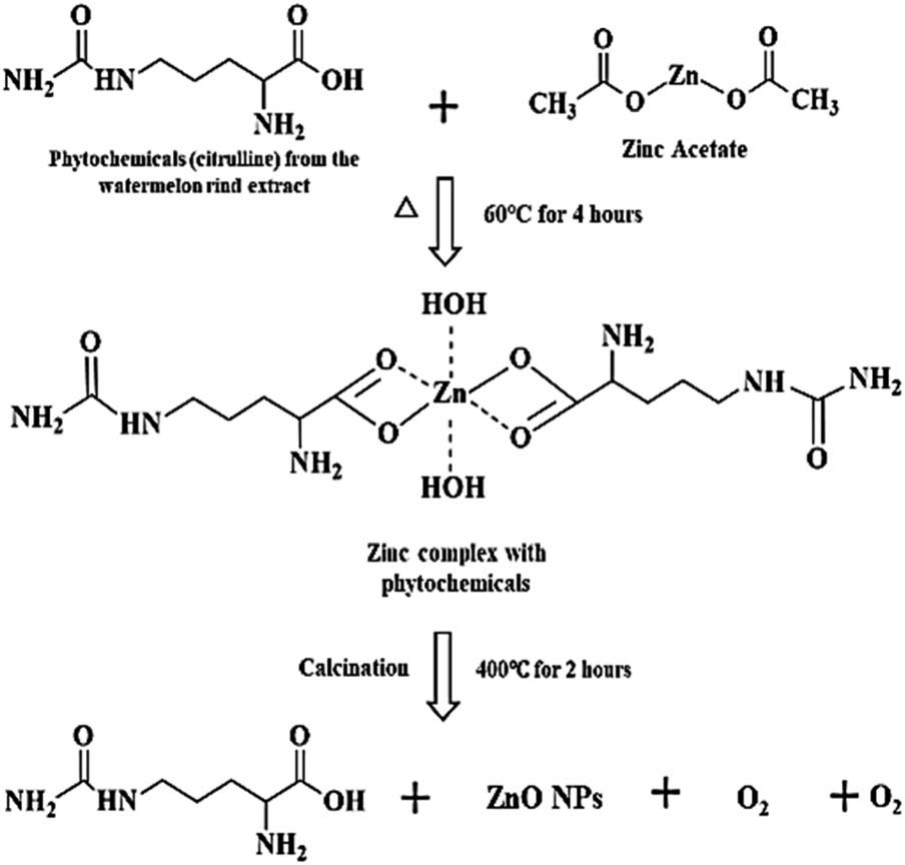
Hypothesized mechanism for the formation of ZPCL from WR extract.

Watermelon rind wastes can be reclaimed, repurposed, or transformed into more sustainable and value-added products such as zinc oxide nanoparticle formation owing to their potent bioactive compounds.^24^ The proposed mechanism of ZPCL green synthesis is described in a two-step process (Fig. 2) involving the formation of the metal complex with the dispensed Zn^2+^ by the interaction of the zinc salt and the WR extract, which on reduction, stabilization, and calcination yield ZnO. The chemical nature and reduction potential of the zinc acetate precursor salt tend to support electron donation. The ionic state of metals can be separated from the anionic component and reduced to the most stable form through chelation with antioxidant phytochemicals, such as citrulline in this instance.^41^ Citrulline is a predominant biomolecule present in WR and has potential chelating properties, which are responsible for the formation of the hydroxide complex.^42^ Due to its functional group's ability to coordinate with metal ions, citrulline can form stable complexes with zinc through chelation, contributing to the stabilization and controlled precipitation of the resulting compound.^43^ As a result, Zn^2+^ easily coordinates with the various phytoconstituents present in the watermelon rind extract through the OH^−^ group, leading to the formation of Zn(OH)_2_. The formation of Zn(OH)_2_ was accompanied by the appearance of a milky white precipitate, marking the completion of the reaction.^44,45^ The synthesized Zn(OH)_2_ is then dried in an oven and subsequently calcined in a muffle furnace to form ZnO, as depicted in Fig. 1. The overall yield of ZPCL was 41.35%.

### 3.2 Characterization techniques

#### 3.2.1 UV-vis spectroscopic analysis

**Fig. 3:**
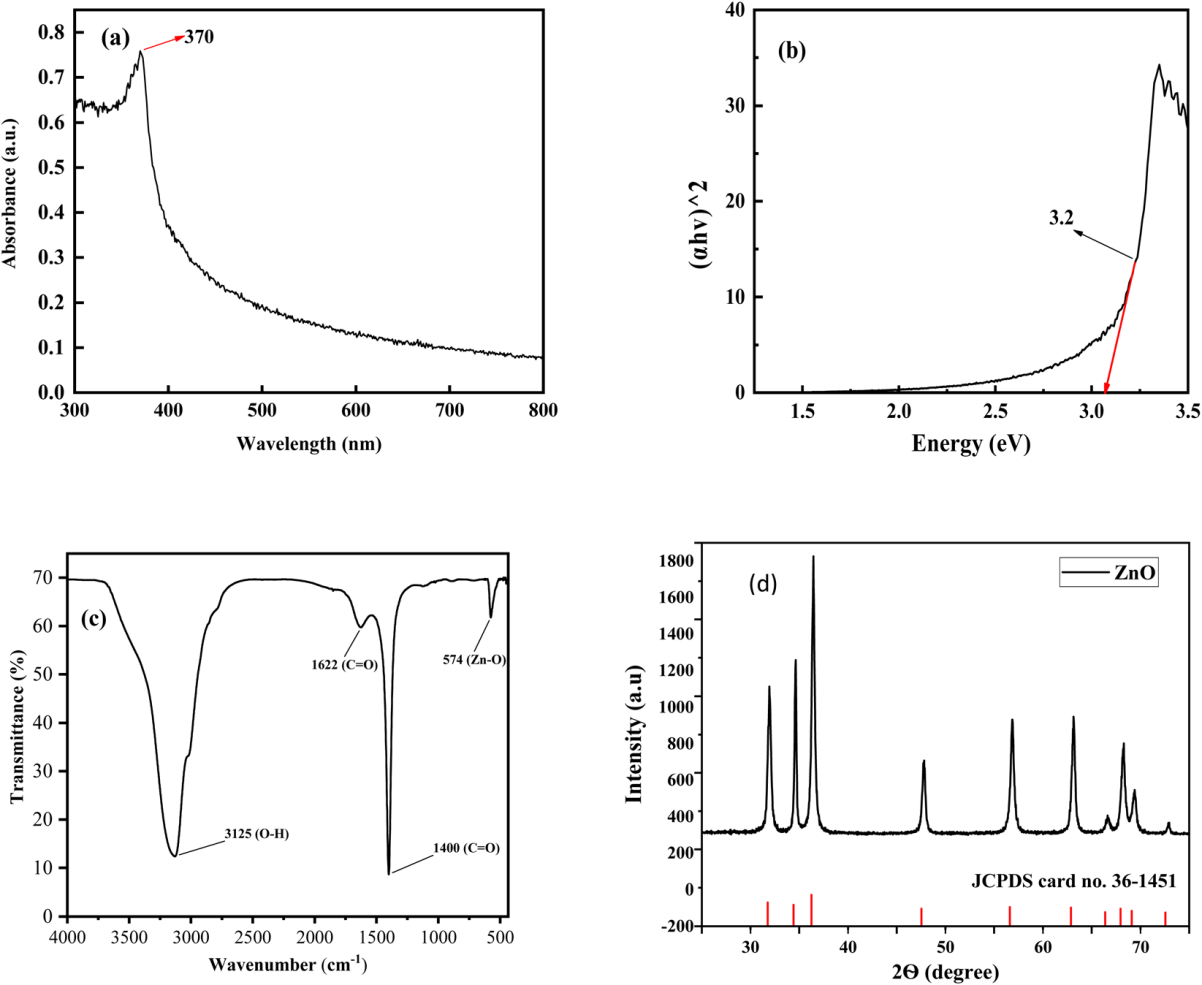
(a) UV-vis spectrum of ZPCL synthesized using *C. lanatus* rind extract, (b) (*αhν*)^2^*versus* the photon energy (*hν*) for ZPCL, (c) FT-IR spectra of nanocrystalline ZPCL, (d) XRD pattern of the produced ZPCL (the bottom bars show the standard ZnO from JCPDS No. 36-1451).

The optical property of the ZPCL was investigated by the UV-vis spectroscopy. The absorption edges, shown in Fig. 3a, were found at 370 nm which is a characteristic of ZnO. In the UV-vis spectrum, there was only one peak that confirmed the purity of the sample. The location of the spectrum depends largely on the particle concentration, size, and shape. The determined UV-vis absorption peak of ZPCL was similar to previously reported literature.^46,47^

The optical band gap was 3.2 eV for the bio-synthesized ZPCL which was determined using the Tauc plot, as depicted in Fig. 3b. The result was in line with the previously reported literature for ZnO NPs.^48^ The wide band gap is evidently attributed to the small particle size of ZPCL, which is further supported by FE-SEM and XRD analysis.

#### 3.2.2 FTIR analysis

The FTIR spectra of ZPCL are shown in Fig. 3c in the range from 4000 to 500 cm^−1^. The spectrum reveals a prominent and intense spectroscopic band at around 574 cm^−1^, attributed to the characteristic Zn–O vibrational mode. This corresponds with previous literature findings, confirming the structure and purity of the synthesized NPs.^49^ The broad absorbance band observed at approximately 3125 cm^−1^ is the result of the stretching vibration modes of the hydroxyl (O–H) group, indicating the presence of alcohol/phenolic bioactive compounds of WR extract.^31,50^ The absorption peak found at 1622 cm^−1^ denotes the C=O stretching and N–H bending vibration of the amine or amide group, which might be derived from the different active constituents of WR extract.^9,51^ The sharp and strong absorption spectrum observed at 1400 cm^−1^ is ascribed to the asymmetric and symmetric stretching vibrations of unidentate acetate species (COO^−^).^52,53^ As evidenced by the above results, the biomolecules present in the WR extract could assist in the reduction and capping of Zn^2+^ and the overall formation of ZnONPs.

#### 3.2.3 XRD analysis

An XRD was performed to determine the average crystallite size, phase purity, and crystallinity of ZPCL. The strong intense and sharp diffraction peaks denoted the excellent crystallinity of the NPs, demonstrating the effective crystal growth of the ZnO particles. Additionally, the noticeable broadening of the diffraction peaks signifies that the synthesized materials exist in the nanoscale range. The XRD patterns of ZPCL revealed distinct diffraction peaks located at 2*θ* values of 31.94, 34.63, 36.45, 47.85, 56.85, 63.14, 66.62, 68.63, and 69.41° as depicted in Fig. 3d. These reflections can be indexed to crystallographic planes (100), (002), (101), (102), (110), (103), (200), (112) and (201), respectively. The pattern identified in the XRD analysis indicates the presence of a hexagonal wurtzite structure of the pure phase of ZPCL, characterized by distinct crystalline peaks. The obtained diffraction results align closely with the previous report^34,54,55^ and are in accordance with the standard peaks displayed by the JCPDS card no. 36-1451. The average crystallite size of ZPCL was determined by utilizing the high-intensity peak, yielding a value of 20.36 nm. This calculation was performed using the Debye–Scherrer equation, specifically applicable to the pure-phase monoclinic crystalline morphology. The percentage of crystallinity was calculated using the peak area, and it was found that the synthesized ZPCL had 77.12% crystallinity.

3 *D* = 0.9*λ*/*β* cos *θ*

This equation gives a relationship between particle size and peak broadening in XRD. Here, *D* = crystalline size. *B* = full width at half maximum (radians). *λ* = X-ray wavelength (0.15406 nm). *θ* = bragg diffraction angle (degrees).

#### 3.2.4 FE-SEM and EDX analysis

**Fig. 4:**
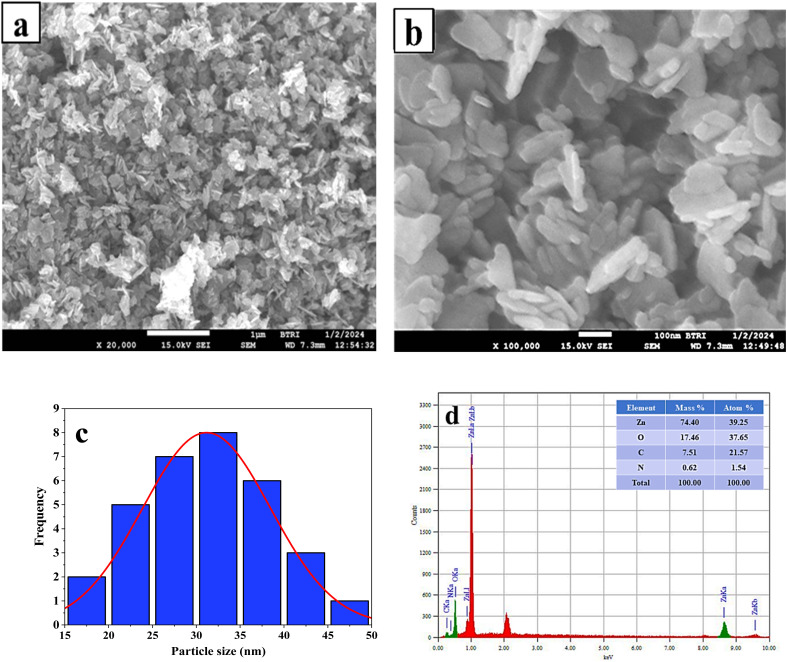
(a) and (b) FE-SEM micrograph of ZPCL synthesized from *C. lanatus* rind extract, (c) histogram of particle size of ZPCL, and (d) EDX spectrograph of ZPCL.

The morphology and particle size of the synthesized ZPCL were investigated through field emission scanning electron microscopy. Fig. 4a illustrates that the initial particles exhibit variable shapes, although most of them are roughly spherical/elliptical shapes and form aggregates. The micrograph clearly shows that the agglomerated clusters are distributed across the surface in a relatively large and random pattern, leaving some empty spaces without a well-defined morphology. This is in accordance with the previously documented ZnO NPs synthesized using the biocomponents of a dry ginger rhizome powder extract, which were reported to have sizes ranging from 23 to 26 nm.^56^Fig. 4b shows the SEM images at higher magnification where particles with a size less than 100 nm were formed. Fig. 4c depicts the histogram of the ZPCL and the size of ZPCL ranged from 17.89 to 44 nm with 31 nm as the average size.

The EDX analysis of the ZPCL sample indicates the presence of the desired phase, with both Zn (74.40%) and O (17.46%) detected in the sample, in which very little impurities can be seen. The appearance of the nitrogen and carbon peaks in the EDX spectrum, as shown in Fig. 4d, is attributed to residual organic contaminants. The chemical analysis through EDX confirms the formation of ZnO which is consistent with the findings reported earlier.^57^

#### 3.2.5 DLS and ζ-potential analysis

**Fig. 5:**
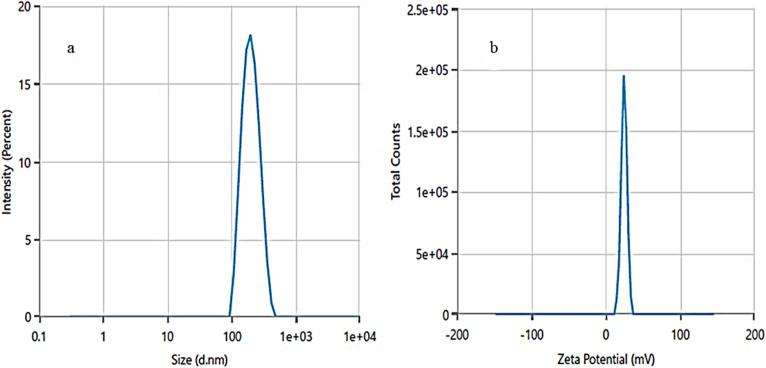
(a) Hydrodynamic size distribution and (b) ζ-potential distribution of ZPCL.

Dynamic light scattering (DLS) and zeta (ζ-) potential analysis were used to determine the hydrodynamic size and stability of ZPCL. DLS analysis indicates a narrow size distribution, with an average hydrodynamic size of ZPCL measured at 193 nm, as illustrated in Fig. 5a. The particle size is polydispersed and bigger than FE-SEM observed particles based on the size distribution graph. This discrepancy might be due to the technique's bias toward measuring larger particles (or even aggregates).^58^ In the study, the ζ-potential of the ZPCL was measured as +24.77 mV (Fig. 5b). The positive sign and magnitude of surface charge suggest stability of the green synthesized ZPCL particles, which ultimately led to their diverse applicability.^58^ Our DLS and ζ-potential results of ZPCL are in agreement with previous reports performed on ZnO NPs using *Elaeagnus angustifolia* leaf extract.^59^

### 3.3 Antimicrobial assay

**Fig. 6:**
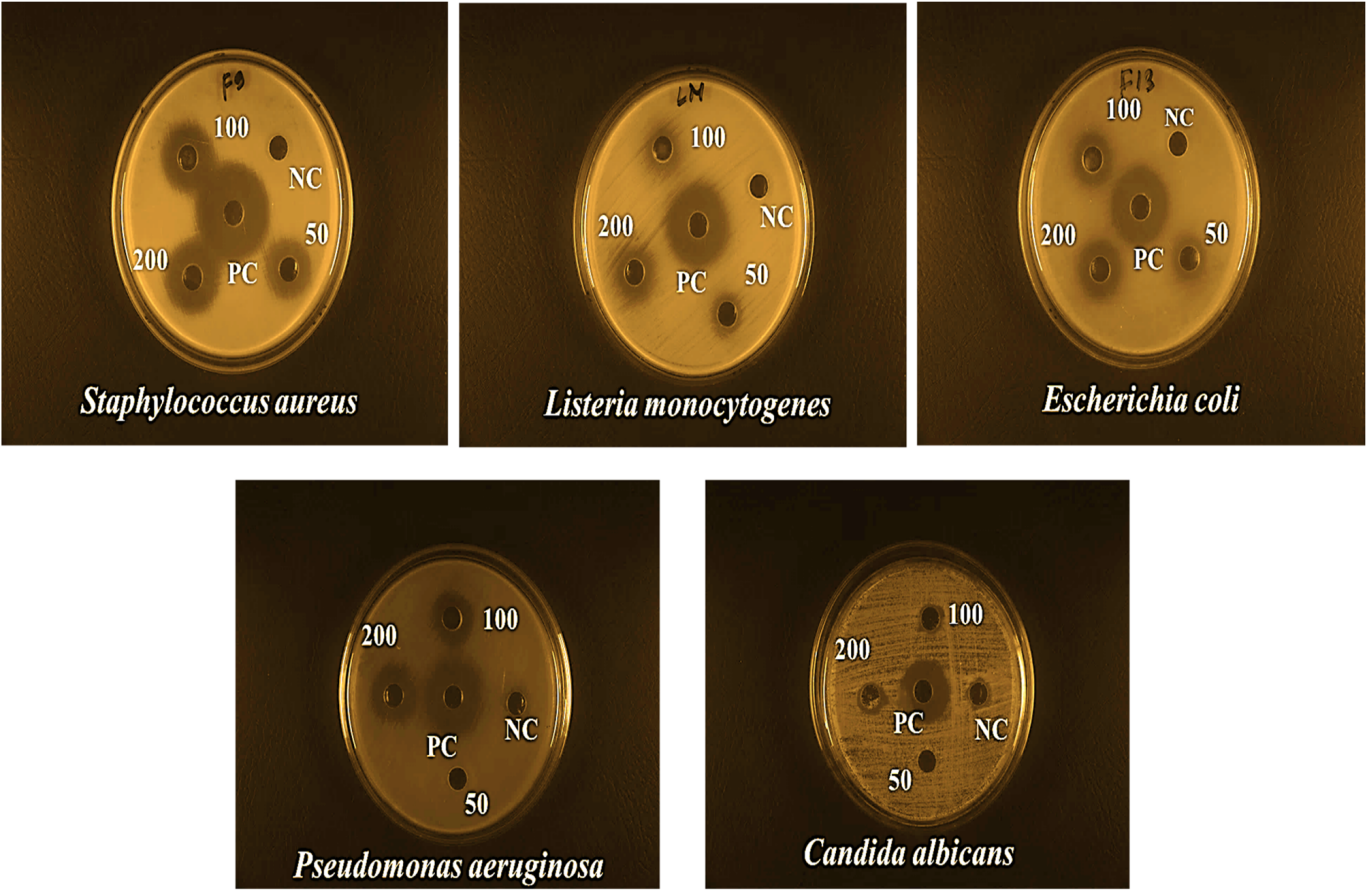
Antimicrobial effects of ZPCL at three different concentrations (50, 100, 200 μg mL^−1^) against *S. aureus*, *L. monocytogenes*, *E. coli*, *P. aeruginosa*, and *C. albicans*.

**Fig. 7:**
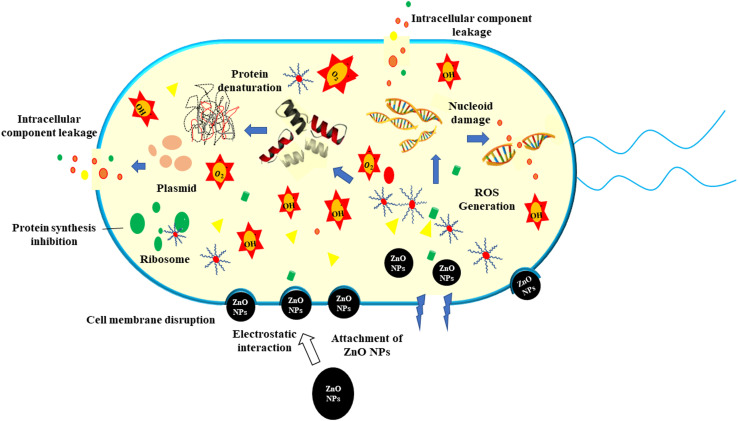
Schematic illustration of the antimicrobial mechanism of ZPCL against pathogenic bacteria.

The current study synthesized ZPCL using watermelon rind and tested their antibacterial and antifungal activity against bacterial pathogens such as *S. aureus*, *L. monocytogenes*, *E. coli*, *P. aeruginosa,* and fungal strain *C. albicans*. It has been shown that biogenic ZnO NPs revealed substantial antibacterial activity in comparison to previous reported studies as depicted in Table 2. The magnitude of the susceptibility of the pathogenic microbial strains to the ZPCL is depicted in Fig. 6. The bactericidal efficacy of ZPCL was concentration-dependent. The study noticed that the inhibitory zone will be greater with increasing concentrations of NPs. Different diameter of the zone of inhibition (ZOI) was observed for the tested microorganisms, which are presented in Table 1. It has been shown that *S. aureus* exhibited the highest ZOI, while *P. aeruginosa* demonstrated the lowest ZOI. This result was consistent with previous findings of MuthuKathija *et al.*, and Chennimalai *et al.*,^34,60^ where Gram-positive bacteria (*S. aureus* and *Bacillus cereus*) were more susceptible to ZnO NPs compared to Gram-negative bacteria. The plausible mechanism of the finding is the distinction between Gram-positive and Gram-negative bacterial cell wall structure. Gram-positive bacterial cell walls are surrounded by thicker peptidoglycan layers and anionic teichoic acids that electrostatically interact with cationic NPs *i.e.*, ZPCL, disrupt bacterial cell membranes leading to cell death.^61^ The cell walls of Gram-negative bacteria are more complex, protected by an outer lipopolysaccharide membrane, and also have thin peptidoglycan layers.^62^ However, it is not fully understood, how these NPs exert their antimicrobial effects. The present study notion that ZnO NPs smaller size and larger surface-to-volume ratio allowed them to interact closely with microbial membranes and triggered the disruption of membrane lipids and proteins, resulting in intracellular components leakage. Moreover, ZPCL can also enter the bacterial cells encouraging microorganisms to emit ROS, causing nucleoid and protein damage, ultimately inhibiting microbial growth and instigating cell death (Fig. 7). These findings elicit the prospects of using ZPCL as a promising therapeutics to combat bacterial infections.

**Table 1:** Inhibition zones (mm) of biosynthesized ZPCL against different pathogenic microorganisms^a^

Pathogenic microorganism	Concentration (μg mL^−1^) and zone of inhibition in mm					
	50	100	200	Chloramphenicol (100)	Carbendazim (100)	50% DMSO
*Staphylococcus aureus* (ATCC 6538)	18 ± 0.04	21 ± 0.03	22 ± 0.01	29 ± 0.23	No	No
*Listeria monocytogenes* (ATCC 13932)	13 ± 0.03	14 ± 0.14	17 ± 0.06	25 ± 0	No	No
*Escherichia coli* (ATCC 25922)	14 ± 0.03	17 ± 0.01	18 ± 0.03	24 ± 0.16	No	No
*Pseudomonas aeruginosa* (ATCC 9027)	No	13 ± 0.14	16 ± 0.03	26 ± 0.06	No	No
*Candida albicans* (ATCC 10342)	No	No	No	No	22 ± 0	No

a The average inhibitory zone diameter (mm) ± standard errors were used to record the results.

**Table 2:** Comparative study on antimicrobial activity of ZnO NPs synthesized from facile green route

Bio-sources	Parts	Mean crystallite size and morphology	Highest concentration of ZnO NPs (μg mL^−1^)	Test organisms	Mean zone of inhibition (ZOI), mm	Ref.
*Citrullus lanatus*	Rind	20.36 nm and irregular spherical/elliptical shape	200	*S. aureus*	∼22	This study
				*L. monocytogenes*	∼17	
				*P. aeruginosa*	∼16	
				*E. coli*	∼18	
				*C. albicans*	0	
Myristica fragrans	Fruit	41.23 nm and spherical or elliptical shape	1000	*E. coli*	15	18
				*K. pneumoniae*	27	
				*P. aeruginosa*	17	
				*S. aureus*	21	
*Pluchea indica*	Leaves	21.9 nm and spherical form	1000	*E. coli*	∼21	63
				*P. aeruginosa*	∼13	
				*E. faecalis*	∼14.9	
				*B. subtilis*	∼24.26	
				*S. aureus*	∼17	
				*C. albicans*	∼20.67	
				*C. neoformans*	∼19	
				*S. aureus*	∼22	
				*E. coli*	∼23	
*Pisonia alba*	Leaves	48 nm and an aggregated, aloe vera leaf form	100	*S. aureus*	∼20.4	34
				*K. pneumoniae*	∼11	
*Terminalia catappa*	Fruit pericarp	12.58 nm and irregular spherical shape	200	*S. aureus*	∼9	8
				*S. pyogenes*	∼19	
				*S. typhi*	∼8	
				*P. aeruginosa*	0	
				*C. albicans*	∼12	
*Punica granatum*	Peel	43 nm and spherical form	200	*S. aureus*	∼17.3	64
				*B. subtilis*	∼17.7	
				*P. aeruginosa*	∼24.7	
				*E. coli*	∼19.3	
				*C. albicans*	∼17.0	

### 3.4 Molecular docking

**Fig. 8:**
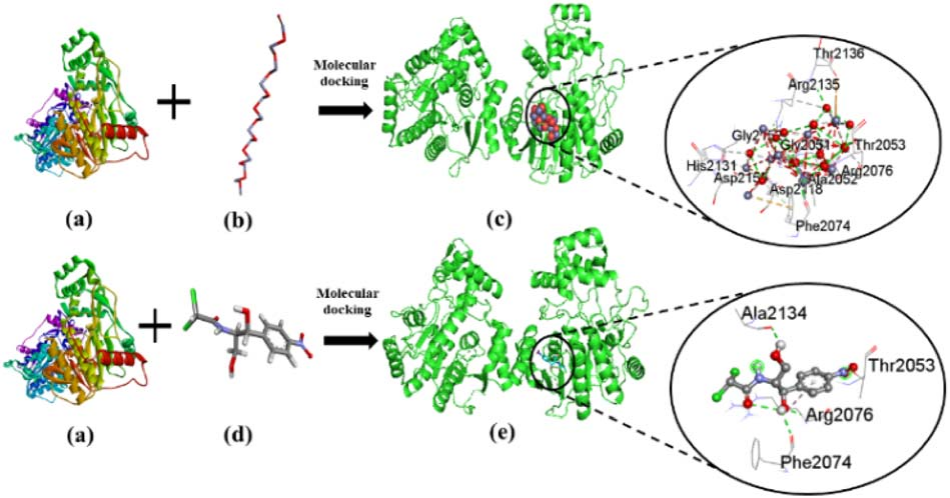
The interaction of ZnO NPs with target protein AusA (PDB: 4F6C) from *S. aureus* and CHL as reference. The left side represents the 3D structure of AusA protein (a), ZnO hexagonal (b), and CHL (d), and the right side represents 3D complex receptor–ligand interaction (c and e).

The current study investigated the binding interaction of ZnO NPs with microbial proteins using molecular docking analysis. The proteins aureusimine biosynthetic cluster reductase domain (PDB: 4F6C) and chloramphenicol acetyltransferases type III (PDB: 6X7Q), are significant for *S. aureus* pathogenicity and chloramphenicol resistance in *E. coli*, respectively, have been recognized as promising targets for novel antibiotics development.^65,66^Fig. 8 depicts the molecular interaction of ZnO NPs and CHL with aureusimine biosynthetic cluster reductase domain (AusA) of *S. aureus* at minimum binding energy (−5.3 kcal mol^−1^ and −6.3 kcal mol^−1^, respectively). Table 3 summarizes the average binding affinity and non-bonding interaction of ZnO and CHL with target proteins. Interestingly, though ZnO NPs demonstrated higher free binding energy compared to CHL, the non-bonding interaction between ZnO and microbial protein was strong. The binding affinity of ZnO and AusA is expedited by the formation of six hydrogen bonds with GLY2051, ALA2052, THR2053, ARG2076, ARG2135, and PHE2074, and also two hydrophobic contacts with HIS2131, and ARG 2135. Moreover, there were three electrostatic and metal acceptor bonds with ASP2118, ASP 2155, THR 2136, and PHE2074, GLY2152, and ARG2076, respectively. Other non-covalent interactions were one-carbon hydrogen, pi–cation, and pi–hydrogen bond with HIS2131 and PHE2074, respectively. Chloramphenicol showed only three conventional hydrogen bonds and one pi-alkyl bond with THR2053, ARG2076, PHE2074, ALA2134, and ARG2076, respectively. The shared residues THR2053, ARG2076, and PHE2074 in the active site of the AusA are involved in the interaction with the targeted drug and facilitated binding affinity. These findings are in line with El-Sayed *et al.*,^67^ who employed the molecular docking approach to assess the inhibitory interaction between Cu-doped ZnO nanomaterials and microbial protein (FabH for *E. coli* and PBPs for *S. aureus*).

**Table 3:** Average binding affinity and non-bonding interaction of ZnO, chloramphenicol, and ascorbic acid ligands with target proteins

Name	Binding affinity (kcal mol^−1^)	Residues in contact	Interaction type	Distance (Å)
AusA_ZnO	−5.3	ASP2118	Attractive charge	4.4323
		ASP2155	Attractive charge	4.74906
		THR2136	Attractive charge	4.35818
		GLY2051	Conventional hydrogen bond	1.83408
		ALA2052	Conventional hydrogen bond	2.9751
		THR2053	Conventional hydrogen bond	2.3122
		ARG2076	Conventional hydrogen bond	2.16048
		ARG2135	Conventional hydrogen bond	2.38461
		ASP2118	Conventional hydrogen bond	3.26245
		PHE2074	Conventional hydrogen bond	3.33502
		HIS2131	Carbon hydrogen bond	3.01464
		ARG 2135	Carbon hydrogen bond	3.53891
		ARG2076	Metal-acceptor	2.51822
		PHE2074	Metal-acceptor	2.59591
		GLY2152	Metal-acceptor	2.65443
		ARG2076	Metal-acceptor	2.51822
		PHE2074	Pi–cation	4.45823
		PHE2074	Pi–donor hydrogen bond	3.96423
AusA_CHL	−6.7	THR2053	Conventional hydrogen bond	2.1141
		ARG2076	Conventional hydrogen bond	2.20881
		PHE2074	Conventional hydrogen bond	2.49831
		ALA2134	Conventional hydrogen bond	2.38287
		ARG2076	Pi-alkyl	4.27255
CAT III_ZnO	−5.3	GLU175	Attractive charge	5.57534
		ASP35	Attractive charge	4.82903
		SER32	Conventional hydrogen bond	2.60588
		LYS33	Conventional hydrogen bond	1.97362
		ARG178	Conventional hydrogen bond	2.49552
		ASN148	Conventional hydrogen bond	2.19395
		LYS171	Conventional hydrogen bond	2.69004
		GLN173	Conventional hydrogen bond	1.97952
		ASN202	Conventional hydrogen bond	3.30825
		GLN173	Carbon hydrogen bond	3.5829
		ASN202	Metal-acceptor	2.44033
		TRP146	Metal-acceptor	2.68793
		TYR172	Metal-acceptor	2.5932
		ASN148	Metal-acceptor	2.66221
		GLN173	Metal-acceptor	2.51983
		GLN205	Metal-acceptor	3.17251
		LYS33	Metal-acceptor	2.69404
		GLN173	Metal-acceptor	2.61805
CAT III_CHL	−6.3	ALA164	Conventional hydrogen bond	2.58967
		HIS138	Conventional hydrogen bond	2.32734
		HIS138	Carbon hydrogen bond	2.44674
		ALA71	Pi-alkyl	4.45805

**Fig. 9:**
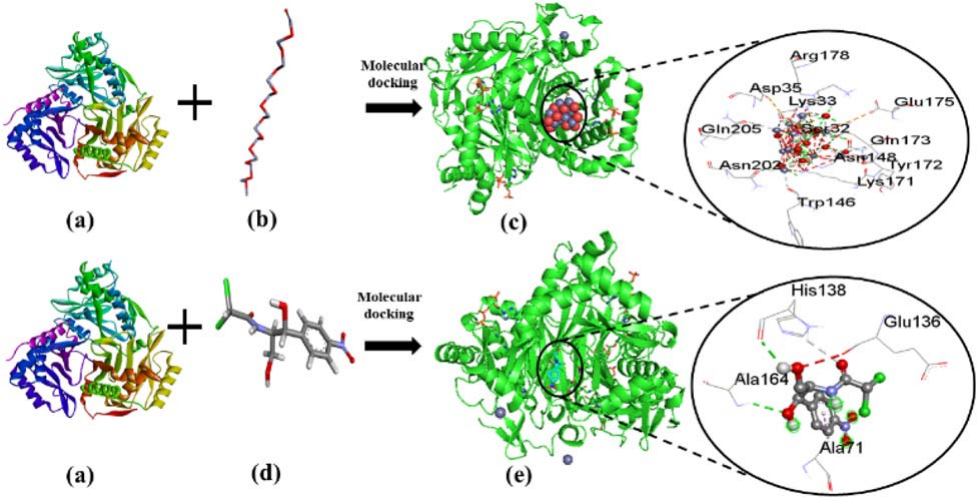
The interaction of ZnO NPs and CHL with target protein CAT III (PDB: 6X7Q) from *E. coli*. The left side represents the 3D structure of CAT III enzyme (a), ZnO hexagonal (b), and CHL (d), and the right side represents 3D complex receptor–ligand interaction (c and e).

Fig. 9 represents the binding mode of ZnO NPs and chloramphenicol acetyltransferases type III (CAT III, 6X7Q for *E. coli*) enzyme, depicting a pictorial representation of their binding interaction. Based on the docking analysis, it can be stated that ZnO NPs have an affinity of −5.3 kcal mol^−1^, compared to Chloramphenicol (–6.3 kcal mol^−1^) with CAT III (Table 3). ZnO NPs formed seven hydrogen bonds with SER32, LYS33, ARG178, ASN148, LYS171, GLN173, and ASN202, and two electrostatic interactions with GLU175 and ASP35. Additionally, hydrophobic interactions included one carbon–hydrogen bond with GLN173 and seven metal acceptor bonds with SER32, LYS33, ASN202, GLN205, TRP146, TYR172, and GLN173. In reference drug, we found only two hydrogen bonds with ALA164, HIS138, and one-pi-alkyl as well as carbon-hydrogen bonds with HIS138 and ALA71, respectively. The active amino acid residues involved in the interaction of ZnONPs with CAT III were TRP146, LYS171, TYR172, ASN202, and GLN205, which play a critical role in stabilizing the ZnO NPs-CAT III complex and facilitating non-covalent interactions between ZnO NPs and CAT III enzyme. In fact, chloramphenicol acetyltransferases type III is an enzyme that confers bacterial resistance to the antibiotic chloramphenicol.^68^ Hence, ZnO NPs could be a promising therapeutic drug target for overwhelming antibiotic resistance. These docking results were consistent with Alam and Álvarez-Chimal *et al.*,^37,39^ findings, who also performed a docking study between ZnO NPs and bacterial proteins (FabH for both G+ve and G−ve strain, and TagF for *S. aureus* and AcrAB-TolC for *E. coli*, respectively) to investigate the inhibitory activity of ZnO NPs. Therefore, our molecular docking results suggested that the inhibition of AusA and CAT III might be a plausible mechanism of action of ZnO NPs antimicrobial activity, and may overcome antibiotic resistance alone or in combination with antimicrobial agents which further needs to be confirmed in *in vitro* and *in vivo* study.

### 3.5 ADMET properties

The AdmetSAR was used to predict pharmacokinetics (human intestinal absorption, Caco-2 cell permeability, blood–brain barrier (BBB), human oral bioavailability, drug metabolism), and acute oral toxicity.^17^ The PRO TOX-II server was used to calculate organ toxicity, metabolism, toxicity endpoint (carcinogen, BBB, clinical toxicity, *etc.*), Tox21-Nuclear receptor signaling pathways (AR, ER, *etc.*), and molecular initiating events (THRα, THRβ, *etc.*).^40^

The ADMET study showed that all the compounds could penetrate the blood–brain barrier, are better absorbed in the gastrointestinal tract, and have greater human oral bioavailability. If a drug has poor bioavailability it may cause a drug candidate's failure in clinical trials even though the drug showed high efficacy in previous *in vitro* and/or *in vivo* tests.^69^ Glycoprotein inhibition assessments were conducted to predict the excretion properties of target compounds. In Table 4, it has been seen that all of the compounds exhibited negative P-glycoprotein inhibition value which indicated P-glycoprotein was not inhibited by any of the compounds rendering no interruption of the absorption, permeability, and retention of the drugs.^70^ Drug metabolism specifically the biotransformation of drug molecules is regulated by various selected cytochrome P450 enzymes (CYPs') *e.g.*, CYP3A4, CYP2C19, CYP1A2, CYP2D6, CYP2E1.^71^ From the tabulated data, negative values of cytochrome enzymes implied that all the compounds showed no inhibitory behavior against these enzymes except chloramphenicol (CYP2C19*I*) suggesting no hindrance to the metabolism of therapeutic drugs. All compounds showed no response towards estrogen receptor binding and androgen receptor binding, except chloramphenicol, which exhibited a response only towards the androgen receptor. This suggests their lack of activity in binding to these receptors resulting in no alteration of the endocrine system.^72^ Besides, all derivatives showed strong inhibition of the human ether-a-go-go-related gene (hERG), which is crucial for the repolarization of the cardiac action potential.^73^ This inhibition did not lead to long QT syndrome or sudden death, highlighting the potential safety of these compounds.

**Table 4:** The predicted ADMET properties of zinc oxide and chloramphenicol using AdmetSAR and PRO TOX-II online server^a^

Name	ZnO	Chloramphenicol
HIA	+0.9769	+0.7284
Caco-2	+0.5834	−0.6684
HOB	+0.7571	+0.8429
BBB	+0.9750	+0.9000
P-Gp*I*/P-Gp*S*	−0.9868/−0.9979	−0.9477/−0.8550
PPB	0.287	0.583
CYP3A4*I*/CYP3A4*S*	−0.9796/−0.8426	−0.8309/−0.6193
CYP2C19*I*	−0.8973	+0.8994
CYP1A2*I*	−0.7620	−0.9046
CYP2D6*I*	−0.9334	−0.9231
CYP2E1	−0.94	−0.99
ERB	−0.9325	−0.6670
ARB	−0.9514	+0.5528
hERG	−0.7986	−0.8548
PPAR-Gamma	−0.9166	−0.8545
nrf2/ARE	−0.79	−0.98
THRα	−0.90	−0.90
THRβ	−0.78	−0.78
NADHOX	−0.97	−0.97
GRB	−0.9034	+ 0.5772
Eye irritation	+ 0.9850	−0.9727
Skin irritation	+ 0.8179	−0.7073
Respiratory toxicity	−0.8	+0.84
Cardiotoxicity	−0.98	−0.53
Hepatotoxicity	−0.97	−0.70
Nephrotoxicity	−0.8	+0.69
Immunotoxicity	−0.99	−0.99
Ames mutagenesis	−0.9800	−0.7700
Mutagenicity	−0.59	+0.63
Cytotoxicity	−0.79	−0.64
Nutritional toxicity	−0.59	+0.57
Biodegradation	+0.6500	−0.6750
AOT	2.034	1.594
LD 50	7950	1500
Toxicity class	VI	IV

a HIA = human intestinal absorption, HOB = human oral bioavailability, BBB = blood–brain barrier, P-Gp*I* = P-glycoprotein inhibitor, P-Gp*S* = P-glycoprotein substrate, PPB = plasma protein binding, CYP3A4*I* = CYP3A4 inhibition, CYP2C19*I* = CYP2C19 inhibition, CYP1A2*I* = CYP1A2 inhibition, CYP3A4*S* = CYP3A4 substrate, CYP2D6*I* = CYP2D6 inhibition, CYP2E*1* = CYP2D6 inhibition, ERB = estrogen receptor binding, ARB = androgen receptor binding, hERG = human ether a-go-go-related gene inhibition, PPAR-Gamma = peroxisome proliferator activated receptor gamma, nrf2/ARE = nuclear factor (erythroid-derived 2)-like 2/antioxidant responsive element, THRα = thyroid hormone receptor alpha, THRβ = thyroid hormone receptor beta, NADHOX = NADH-quinone oxidoreductase, GRB = glucocorticoid receptor binding, AOT = acute oral toxicity (mol kg^−1^), LD 50 = lethal dose 50 (mg kg^−1^).

Based on toxicity profiles assessed using the PRO TOX-II server, compounds were screened for respiratory toxicity, cardiotoxicity, hepatotoxicity, nephrotoxicity, immunotoxicity, mutagenicity, cytotoxicity, and nutritional toxicity as shown in Table 4. ZnO was found to be mostly nontoxic and is considered a safer alternative to chloramphenicol, which exhibited some detrimental impact on metabolic processes rather than inhibiting it. Thus, ZnO emerges as a more acceptable candidate for future drug development. According to the predicted toxicity class, ZnO was classified as class VI whereas chloramphenicol was categorized as class IV. Given that a lower class corresponds to higher toxicity, ZnO was considered less toxic compared to the other compounds.

### 3.6 Photocatalytic activity

**Fig. 10:**
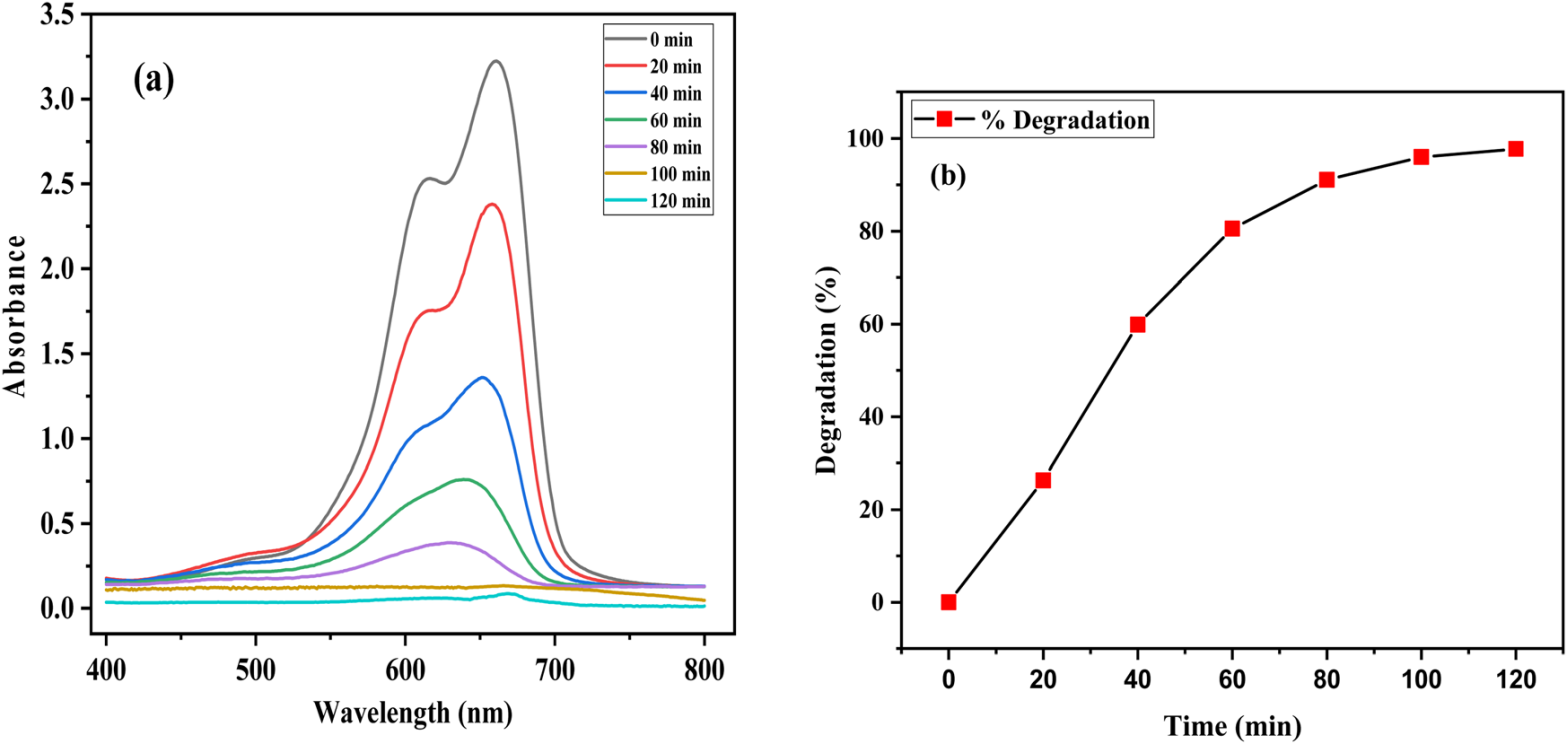
(a) UV-vis absorbance spectra of the photocatalytic decomposition of methylene blue by ZPCL and (b) percentage degradation of methylene blue with ZPCL at different time intervals.

**Fig. 11:**
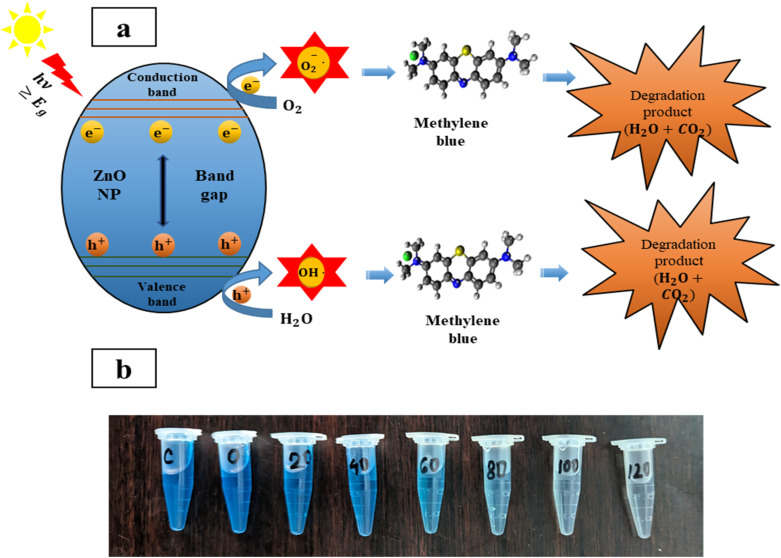
(a) Schematic illustration of photocatalytic decomposition mechanism of methylene blue, and (b) visual decolorization of methylene blue by ZPCL photocatalyst.

Photocatalytic degradation of MB (20 ppm) dye was investigated by the biofabricated ZPCL (25 mg) under solar light irradiation at different time intervals 20, 40, 60, 80, and 100 min. The dye decomposition was confirmed by the decolorization of the dye as shown in Fig. 11b while the catalytic activity of the nanoparticles was evaluated based on this change in intensity around 660 nm. Fig. 10a demonstrated that UV-visible absorption spectra of MB dye steadily decreased with increasing illumination time and almost disappeared at 120 min. In the initial 20 min, the degradation was 26.3%, while with prolonged exposure to sunlight, the degradation of the dye reached a maximum of 99.02% at 120 min as denoted in Fig. 10b. In addition, the current study has showed distinctive efficiency in the rapid degradation of MB dye by using biosynthesized ZnO NPs in comparison to previous reported literature as indicated in Table 5.

**Table 5:** Comparative study on photocatalytic performance of zinc oxide nanoparticles synthesized by various green source^a^

Green source of ZnO NPs	Organic compound	Concentration of dye (ppm)	Catalyst dosage (mg)	Light source	Reaction time (min)	Efficiency (%)	Ref.
*Passiflora foetida* peel extract	MB, RhB	10	100	Sunlight	70	93.25%; 91.06%	74
*Myristica fragrans* fruit extract	MB	20	25	UV	140 min	88%	18
*Punica granatum* peel extract	MB	10	20	UV	210	93.4%	64
*Aloe vera* leaf extract	MG	10	10	Sunlight	240	85	75
*Thymus vulgaris* leaf extract	MB	10	120	UV	30	96	76
Rambutan peel extract	MO	10	100	UV	120	83.99	77
*Citrus reticulata* blanco peel/extract	AG	50	50	UV	90	94	78
*Moringa oleifera* peel extract	CV	1	5	UV	70	94	79
*Lantana Camara* flower extract	MB	20	20	UV	75	96	80
Durian rind extract	Sulfanilamide	10	10	Sunlight	180	96.70	81
Jujube fruit extract	MB,ECBT	100	15	Sunlight	300	>85	82
*Citrullus lanatus* rind extract	MB	20	25	Sunlight	100	99.02	Present work

a MB = methylene blue, MG = malachite green, RhB = rhodamine B, MO = methyl orange, AG = acid green, CV = crystal violet, ECBT = eriochrome black-T.

The study revealed the association between the decomposition process and irradiation time, hence, it is crucial to know the mechanism behind this degradation process. Initially, MB dye is adsorbed on the surface of ZPCL due to its high surface area-to-volume ratio. Upon sunlight irradiation of the catalyst (ZPCL) electrons (e^−^) situated in the valence band get excited and transition into the conduction band due to the wide band gap energy of ZnO NPs. This wide band gap energy facilitates this transition of e^**−**^ from valence band by supplying sufficient energy to overcome the gap. During this transition, an equal number of holes (h_vb_^+^) and electrons (e_cb_^−^) are created, leading to the formation of electron–hole pairs (e^−^/h^+^) and initiating a series of reactions. The e^−^ located in the conduction band will cause a reduction of the dissolved oxygen by converting it into superoxide anion radicals (O_2˙_˙^−^). These radicals further react with H^+^ and convert them into hydroperoxyl radicals (HO_2˙_), which then undergo further conversion into hydrogen peroxide and oxygen. The holes in the valence band will too react with H_2_O by converting it into H^+^ and OH^.^. These ROS, such as hydroxyl radicals, are strong oxidizing agents and play an important role in decomposing dye molecules into smaller and less detrimental substances such as H_2_O and CO_2_.^16,18,83^ As the ZPCL are exposed to sunlight for a longer duration, more photons are absorbed by the nanoparticle, leading to an increase in the emergence of ROS on the nanoparticle surface. As a result, more dye molecules are subjected to the photocatalytic degradation process by these ROS, resulting in a greater reduction in dye concentration and an increase in percent dye degradation. Fig. 11a illustrates the mechanism of photocatalytic degradation of MB dye and the corresponding photocatalytic reaction process can be described by eqn (4)–(9).

4 ZnO + *hν* → e_cb_^−^ + h_vb_^+^

5 O_2_ + e_cb_^−^ → O_2˙_˙^−^

6 H_2_O + h_vb_^+^ → OH˙ + H^+^

7 O_2˙_˙^−^ + H^+^ → HO_2˙_

8 HO_2˙_ + HO_2_ → H_2_O_2_ + O_2_ → 2OH˙ + O_2_

9 OH˙ + dye → Degradation product (H_2_O + CO_2_)

### 3.7 ZPCL reusability

**Fig. 12:**
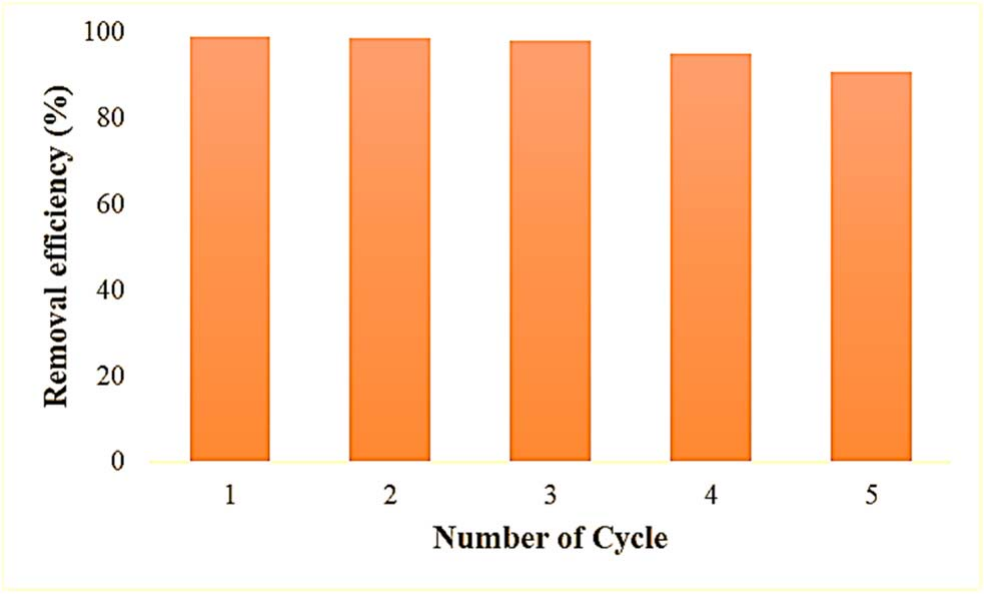
Reusability potential of ZPCL after five consecutive cycles of MB degradation.

**Fig. 13:**
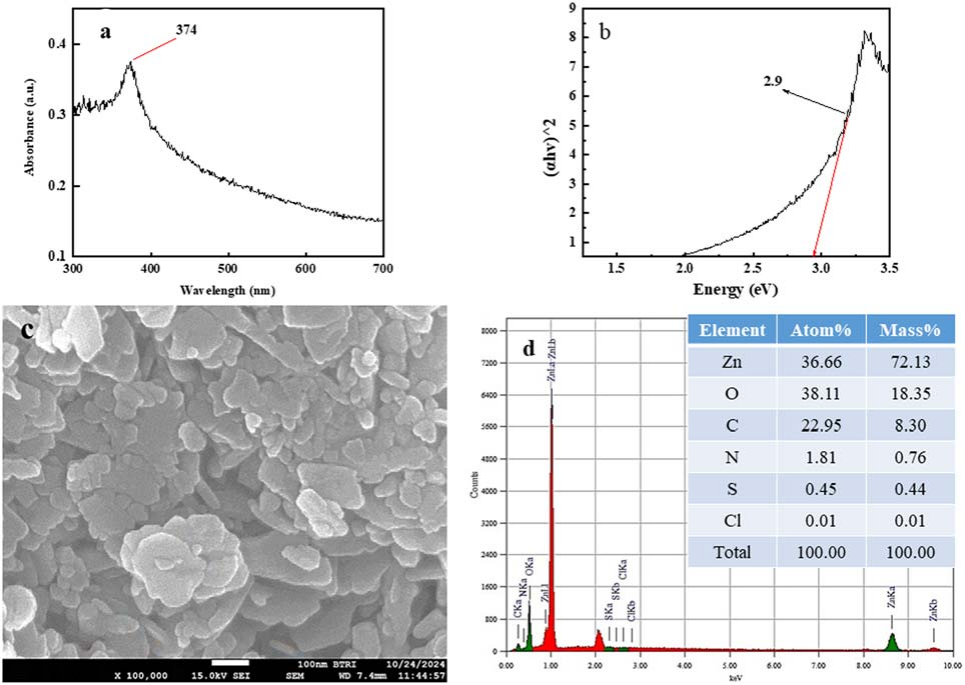
ZPCL characteristics after photocatalytic cycling experiment using UV visible spectroscopy, FE-SEM and EDx analysis.

Recovery and reusability are critical factors in selecting a realistic and economical catalyst for the remediation of hazardous dyes. The reusability potential of ZPCL (25 mg) was explored for five consecutive cycles of 50 mL MB (20 ppm) dye degradation under solar light irradiation (Fig. 12). After each cycle, catalysts (ZPCL) were washed several times with DI and ethanol then dried in an oven at 80 °C for 10 h. It was observed that for the first three cycle MB dye was almost decomposed (∼99–98%) and the next two cycle dye degradation was slightly decreased from ∼95% to ∼90% after each 120 min cycle. The plausible explanations for this insignificant decline in photocatalytic activity might be due to the agglomeration of particles and the accumulation of dyes on the catalyst surface, which can reduce the availability of active sites, reducing their accessibility for further photocatalytic reactions. The Fig. 13 might shed light on the aforementioned mechanism, as it is depicted that ZPCL showed reduced band gap energy (b), agglomeration of particles (c), and the presence of some extra elements such as C, N, S, *etc.* (d) compared to the before photocatalytic cycling experiment. As a result, the overall efficiency of the photocatalytic process is hindered because fewer active sites are available to facilitate the degradation of the dye. In addition, our findings were consistent with Boutalbi *et al.*, 2023 and Tabet *et al.*, 2024 studies.^84,85^ Overall, the findings suggest that biogenic synthesized ZPCL photocatalysts have the potential to be used repetitively in the process of MB dye degradation.

## 4 Conclusion

This research work is focused on a facile green route to synthesize ZnO NPs using *Citrullus lanatus* waste peel extract that proved to be an alternative eco-friendly method. The synthesized ZPCL was confirmed by various physicochemical characterization techniques. The biosource-waste-mediated ZPCL showed noteworthy antimicrobial activity that was further corroborated by the molecular docking simulations. According to *in vitro* and *in silico* analysis, ZnO NPs hold promise to combat bacterial infection owing to their potential binding affinities to these target proteins. Thus, our findings suggest that the biogenic ZnO NPs have the potential as a distinctive therapeutic agent for treating disease induced by bacterial infection and would be a safe drug based on ADMET prediction. The potential of our work also lies in environmental remediation by degrading detrimental methylene blue dye. This study revealed ZPCL as an efficient adsorbent capable of decomposing more than 99% methylene blue dye under photo-irradiation, demonstrating their potential as an ideal candidate to alleviate the issues arising from dye pollution. Thus, the findings have paved the way to utilize this green and economically feasible method for the synthesis of nanoparticles from waste peel extract in addressing various biomedical and environmental issues. However, further studies are warranted to confirm the biomedical applications of ZnO nanoparticles at both *in vitro* and *in vivo* levels and to evaluate their capacity for degrading more dyes and other pollutants at domestic and industrial scales. Future research might focus on the optimization of the synthesis and/or synergies with other antimicrobial agents to scale up the fabrication that could improve their overall performance in environmental and biomedical applications.

## List of abbreviations

ADMET: Absorption, distribution, metabolism, excretion, and toxicity
AusA: Aureusimine biosynthetic cluster reductase domain
*C. lanatus*
: *Citrullus lanatus*
CAT III: Chloramphenicol acetyltransferases type III
CHL: Chloramphenicol
Deionized water: DI
DMSO: Dimethylsulfoxide
EDX: Energy dispersive X-ray spectroscopy
FE-SEM: Field emission transmission electron microscopy
FTIR: Fourier transform infrared spectroscopy
HO_2˙_: Hydroperoxyl radicals
MB: Methylene blue
MHA: Muller–Hinton agar
NPs: Nanoparticles
O_2˙_˙^−^: Superoxide anion radicals
ROS: Reactive oxygen species
WHO: World Health Organization
WR: Watermelon rind
XRD: X-ray diffraction
Zn(OH)_2_: Zinc hydroxide
ZnO: Zinc oxide
ZOI: Zone of inhibition
ZPCL: Zinc oxide nanoparticles synthesized from *Citrullus lanatus*

## Data availability

All the data used in this article will be available in the repository of BCSIR (Bangladesh Council of Scientific and Industrial Research), Bangladesh.

## Author contributions

Hajara Akhter: conceptualization, methodology, data curation, formal analysis, investigation, funding acquisition, original draft writing, review & editing; Susmita Sarker Ritu: investigation, data curation, original draft writing, formal analysis, visualization, review & editing; Shahariar Siddique: resources, data curation, investigation; Fariha Chowdhury: methodology, formal analysis, validation; Rehnuma Tasmiyah Chowdhury: visualization, validation; Samina Akhter: review & editing, Mahmuda Hakim: supervision, project administration, review & editing.

## Conflicts of interest

The authors declare no competing interests.
